# Commentary: Anaerobic Contribution Determined in Swimming Distances: Relation With Performance

**DOI:** 10.3389/fphys.2018.00507

**Published:** 2018-05-07

**Authors:** Ricardo J. Fernandes, Victor M. Reis, Cosme F. Buzzachera

**Affiliations:** ^1^Faculty of Sport, Centre of Research, Education, Innovation and Intervention in Sport, University of Porto, Porto, Portugal; ^2^Porto Biomechanics Laboratory, University of Porto, Porto, Portugal; ^3^Research Center in Sports Sciences, Health Sciences & Human Development, CIDESD, Vila Real, Portugal; ^4^Department of Physical Education, North University of Paraná, Londrina, Brazil

**Keywords:** anaerobic capacity, oxygen uptake, lactate, swimming, backward extrapolation

In their article, Campos et al. (2017) concluded that the highest values of anaerobic contribution in competitive swimming occur at the 200 and 400 m distances and are decisive in performances below 400 m. This is an important contribution regarding the energy balance of different competitive events and subsequent training prescription. It is especially important because “swimmers spend a long training time improving specific metabolisms.” We fully agree since elite swimmers are engaged in two (or more) daily training sessions, 6/7 days a week, typically performing 10,000–20,000 m/day (Chatard and Stewart, 2011). This implies the repetition of the same gestures thousands of times per day, overloading muscles, joints and tendons, developing painful overuse injuries impeditive of continuing practicing (Serra et al., 2017).

However, we found some imprecisions in their manuscript, preventing readers better understanding the main message. It begins saying that “the aerobic contribution seems to be easily calculated by the integral of oxygen consumption (VO_2_) during the effort” and “the determination of the anaerobic contribution is complex.” In fact, although VO_2_ uptake assessment during swimming is not new (Sousa et al., 2014), it requires cumbersome procedures and equipment, and specialized personal. Moreover, swimmers need to be followed along the pool (swimming flumes are scarce) using open-circuit metabolic carts or portable gas analyzers transported on a chariot (Fernandes et al., 2003) or on a stick/cable over the water (Sousa et al., 2014; De Jesus et al., 2015). So, even if mathematically the VO_2_ integral is “easily calculated,” the experimental data setup is complex and very demanding (Chaverri et al., 2016), probably explaining the preference of Campos et al. in assessing VO_2_ during the recovery period after exercise.

Furthermore, even if a more hydrodynamic, ergonomic, and comfortable snorkel generation is available (Baldari et al., 2013), it still does not allow diving and tumble turning, leading to lower velocities comparing to unimpeded swimming (Barbosa et al., 2010; Ribeiro et al., 2016). However, we disagree that this apparatus “clearly disrupts the motor pattern,” as there are no evidences of relevant technical modifications. Swimming velocity changes are not due to general kinematics or swimming efficiency alterations (Barbosa et al., 2010) and similar physiologic and biomechanical values were observed when swimming with/without snorkel at the same velocity (Pinna et al., 2012; Ruiz-Teba et al., 2015). Defending that the respiratory snorkel does not allow “making undulations” and “side respiration impossible during the effort” are not valid arguments since: (i) front crawl, the technique mostly used in VO_2_ uptake studies, does not include such undulating movements; (ii) breathing to the side constraints higher speeds, leading swimmers to avoid it in sprint events; (iii) snorkels are frequently used in swimmers daily workouts for correcting asymmetries (Seifert et al., 2008). So, even if direct oximetry has the advantage of allowing gas exchange measurements during swimming, it is a hard task due to the environmental characteristics and the equipment constraints.

We also disagree that “the most accepted method to estimate anaerobic contribution is the accumulated oxygen deficit” (AOD; see Reis et al., 2010), since blood lactate concentrations, as the end product of glycolysis, have been used since the 1970s for evaluating swimmers and controlling training process (e.g., Mader et al., 1978). Inclusively, in the 1980/1990s, due to the development of portable battery-operated automated analyzers, a growing number of researchers/coaches started testing during training and, even, in competition. Therefore, lactate has been often used as the indicator of anaerobic (lactic) metabolism, particularly through this equation (Zamparo et al., 2011):

$$\begin{array}{l} \text{AnL}={\left[\text{L}{\text{a}}^{-}\right]}_{net}\cdot \beta \cdot \text{M} \end{array}$$

where the anaerobic lactic contribution for the overall energy cost is obtained multiplying lactate net accumulation after exercise, the energy equivalent for its accumulation in the blood and the subject mass. Those authors also proposed determining the anaerobic alactic energy contribution through the phosphocreatine (PCr) splitting in the contracting muscles using this equation:

$$\begin{array}{l} \text{AnAL}=\text{PCr}\cdot \left(1-e^{\frac{-t}{\tau }}\right)\cdot \text{M} \end{array}$$

where *t* is the time duration, τ is the time constant of PCr splitting at work onset, M is the subject mass and PCr reflects its concentration at rest. Thus, in opposition to the AOD method (a rather difficult to apply procedure), this methodology allows obtaining more easily and independently both anaerobic lactic and alactic contributions, being inaccurate to say that this energy balance “is frequently ignored in swimming.”

Campos et al. (2017) referred some of the AOD limitations but, for assessing the anaerobic alactic contribution *per se*, they have determined the fast component of post-exercise VO_2_ through the backward extrapolation technique, allowing “maintaining the ecological validity of measurements and increasing the results applicability.” That methodology has been severely criticized, since it is an indirect technique and includes errors derived from a delay at the onset of VO_2_ recovery (Pinna et al., 2012; Chaverri et al., 2016). We know from experience (Laffite et al., 2004) the difficulty of assuring that swimmers successfully hold their breath completely at the swim end, especially when exercise is all-out and supramaximal. Backward extrapolation overestimates swimming VO_2_ (Lavoie et al., 1983) and a forced apnea in the last moments of exercise will likely induce an augmented expiration, rising post-exercise VO_2_ to values that does not represent the true recovery baseline. So, although easily applied in swimming, it could lead to significant inaccuracies, related to the time necessary for putting the face mask, the high possibility of leaks, the many potential errors of the breath-by-breath analysis and the logarithmic back extrapolation requirement (Sousa et al., 2014).

This technique was proposed for estimating alactic energy at high-intensity short exercise bursts that might not be applicable to swimming races over 100 m. It is possible that the Campos et al. observation of larger alactic energy in longer events is due to methodological imprecisions in non-really anaerobic all-out exercises. It has been established that EPOC does not merely represents anaerobic exercise bioenergetics, but also reflects overall return to homeostasis (Asmussen, 1946). The Campos et al. alactic energy values at the 50 and 200 m events are within the literature physiological limits but, even though varying considerably between subjects, the same swimmers in different events should not have presented such differences. The theoretical model herein states that alactic energy is depleted within the first 30 s of exercise and is replenished only when exercise stops. As this was not proven to occur during supramaximal exercise itself, the longer the event, the lower mean exertion, with alactic energy values decreasing or maintaining, but not rising.

Results herein imply that swimming 200 m involve a larger amount of alactic energy comparing with 50 m all-out, which is not consistent with exercise physiology general knowledge. We disagree with the authors justifications that: (i) this “may be explained by the short effort time … too short to increase VO_2_ to the same level as the other distances,” as there is no rationale considering that a shorter event could have less alactic energy production due to the oxidative metabolism inertia and (ii) as 50–400 m are performed “above their critical speed … it does not enable recovery of creatine phosphate (Jones et al., 2008),” since these authors compared predominantly aerobic knee-extension exercise, very different from the analyzed swimming trials. We must insist that there is no rationale to sustain phosphate-pool energy replenishment during all-out competitive swimming exercise.

Thus, without refuting the relevance of their study, we wonder if the data presented could be negatively influenced by the methodologies used. Although some findings are in accordance with the literature and coaches believes, as the lower total anaerobic contribution in the 800 m and the highest values in the 200 and 400 m (e.g., Bonifazi et al., 1993; Vescovi et al., 2011), the lower anaerobic alactic contribution in the shorter event (50 m) is hard to accept (see Capelli et al., 1998; Gastin, 2001 for exercise in general and swimming in particular, respectively). In fact, we have observed a VO_2_ amplitude of 2.7 ± 0.7 L/min after swimming at a ~400 m intensity, which is lower than the value found by the authors for the 100, 200, and 400 m. We remember that, despite finding good VO_2_ backward extrapolation method agreement, it was concluded before that, to ensure its validity, short-duration exercises and supramaximal intensities should be avoided (cf. Chaverri et al., 2016). In the future, to overcome these short-comings and limitations, we suggest a data comparison between the referred methods. We would be delighted to collaborate.

## Author contributions

RF has fully reviewed and criticized the original article, drafted the commentary, reviewed and approved the final manuscript; CB and VR have also reviewed and criticized the original article, assisted in drafting the commentary, reviewed and approved the final manuscript.

### Conflict of interest statement

The authors declare that the research was conducted in the absence of any commercial or financial relationships that could be construed as a potential conflict of interest.
